# Research progress on novel bionic cryoprotectants in gamete and embryo cryopreservation

**DOI:** 10.3389/fvets.2026.1797831

**Published:** 2026-04-16

**Authors:** Lidan Yang, Zesen Yang, Ximo Guo, Yunchang Zheng, Shujing Li, Weihua Du, Shijie Li

**Affiliations:** 1 College of Life Sciences, Hebei Agricultural University, Baoding, China; 2 Institute of Animal Sciences, Chinese Academy of Agricultural Sciences, Beijing, China; 3 Hebei Tianquan Elite Dairy Ltd., Luquan, Shijiazhuang, China

**Keywords:** bionic cryoprotectant, cryopreservation, embryos, gametes, ice crystals

## Abstract

Cryopreservation technology has become a primary method for preserving animal genetic resources in the biomedical field. It achieves long-term storage by placing gametes or embryos in an ultra-low-temperature environment, enabling them to retain biological activity after thawing. However, the cytotoxicity issues associated with traditional cryoprotectants limit their application, making the development of non-toxic and highly effective novel cryoprotectants a current research priority. This review systematically reviews the development history and principles of cryopreservation technology, and summarizes the classification and mechanisms of action of cryoprotectants, with a particular focus on five types of novel cryoprotectants featuring bioinspired structural characteristics. Their ice-suppression performance and mechanisms of action are analyzed. These protectants possess structures similar to those of natural antifreeze substances and exhibit low toxicity, thereby demonstrating higher cryopreservation efficiency, and are thus referred to as novel bioinspired cryoprotectants. Furthermore, this review examines the potential applications of the aforementioned protectants in the cryopreservation of animal gametes (sperm, oocytes, and embryos), offering new insights into expanding the use of bioinspired cryoprotectants in the preservation of animal genetic resources.

## 1 Introduction

The cryopreservation of gametes and embryos involves maintaining sperm, oocytes, and embryos at ultra-low temperatures to slow or halt cellular metabolism and division. Notably, it enables resumed development upon return to physiological temperatures (1). This technique has evolved into a vital tool in life sciences, offering broad applications in livestock production, animal genetic resource conservation, and human fertility preservation. Research on gamete cryopreservation began in 1776 when Italian physiologist Spallanzani and colleagues reported that snow could preserve sperm viability (2). A major breakthrough in semen cryopreservation technology occurred in 1949, when Polge et al. (3) demonstrated that a glycerol (GLY)-containing diluent could maintain pre-freezing viability in frozen chicken semen, establishing a milestone in cryobiology. Research on oocyte cryopreservation commenced in 1976. The following year, Whittingham (4) achieved the first successful cryopreservation of mouse oocytes, which yielded offspring after fertilization. Embryo cryopreservation was first reported in 1972 when Whittingham et al. (5) pioneered the slow-freezing method, enabling mouse embryos to produce offspring after thawing and transfer. Subsequently, cryopreservation of embryos from cattle (6), rabbits (7), rats, sheep (8), horses, humans, and pigs has been achieved successfully. On note, the number of frozen bovine embryos transferred surpassed that of fresh embryos for the first time in 2017, signifying the widespread global adoption of cryopreservation technology (9).

Despite the rapid advancements in cryopreservation technology, it faces numerous challenges, including the toxicity of traditional cryoprotective agents (CPAs) to gametes and embryos, and various forms of damage that occur during freezing. Damage during freezing includes ice crystal injury caused by high intracellular water content, supercooling shock, mitochondrial damage, cytoskeletal disruption, and zona pellucida injury. These forms of damage significantly reduce fertilization, pregnancy, and hatching rates in large animal oocytes. Consequently, the use of novel, low-toxicity CPAs, including antifreeze proteins (AFPs), nanomaterials, oligomers, and polysaccharides, has become more popular in cryopreservation research. These protectants possess structures similar to those of natural antifreeze substances and exhibit low toxicity, thereby demonstrating higher cryopreservation efficiency, and are thus referred to as novel bioinspired cryoprotectants. For instance, poly(L-proline) elevated the cryopreservation survival rate of human oocytes to 99.11% (10). In another study, adding pine needle AFPs to cryopreservation diluents increased the fertilization rates of frozen chicken sperm by more than 80% (11). Moreover, adding 10?5 mol/L melatonin to the vitrification-thawing system mitigated cryoinjury in mouse morulae. Post-thaw embryos exhibited a blastocyst rate (96.30%), hatching rate (74.10%), blastocyst cell number (94.93 ± 2.13), and inner cell mass/total blastocyst cell ratio (28.51% ± 0.94%) that were not significantly different compared to the corresponding rates of fresh embryos (12). This review first provides an overview of the cryopreservation methods and principles for different gamete materials (sperm, oocytes, and embryos). The functional characteristics of traditional cryoprotectants are summarized, and five types of novel bioinspired cryoprotectants—antifreeze proteins, nanomaterials, synthetic polymers, polysaccharides, and deep eutectic solvents—are highlighted, along with their mechanisms of action. Furthermore, the application potential of these cryoprotectants in the cryopreservation of livestock germplasm resources is explored, aiming to promote the in-depth application of efficient, low-toxicity, bioinspired cryoprotective strategies in this field.

## 2 Methods and principles of cryopreservation

### 2.1 Methods and principles of sperm cryopreservation

Mammalian sperms are small in volume, have a large surface area, contain very little cytoplasm compared to other cells. The freezing process causes water to permeate outward from the sperm cell, leading to severe dehydration of the sperm cell. In addition, the formation of ice crystals can damage the sperm plasma membrane and alter the osmotic pressure of the surrounding environment, significantly reducing sperm quality after thawing. Therefore, the principle of sperm cryopreservation is to rapidly freeze or thaw them under the influence of CPAs, avoiding the ice-crystal-forming temperature range of −60 °C to 0 °C whenever possible. Notably, achieving vitrification is essential for facilitating long-term sperm preservation.

Cooling and equilibration are critical factors that affect cryopreservation efficacy (13, 14). An appropriate cooling rate allows sufficient time for intracellular water to efflux, preventing ice crystal formation. Equilibration facilitates the permeation of cryoprotectants, thereby enhancing cold tolerance (15, 16). Studies postulate that optimal equilibration conditions vary among species. For instance, cooling Hu sheep semen for 2.5 h, followed by equilibration with GLY for an additional 2.5 h, significantly improved sperm characteristics (17). Equilibrating equine sperm for 2–4 h (16) and human sperm at 4 °C for 10 min (18) effectively enhanced post-thaw motility. Porcine sperm exhibit high sensitivity to cold shock; even after 1.5 h of equilibration, damage persists, although acrosome structure remains intact (19, 20). Therefore, further exploration is needed to reduce sperm damage during equilibration. Currently, slow freezing, rapid freezing, and vitrification are the primary methods employed. Slow freezing remains the most widely applied method for sperm cryopreservation. However, the post-thaw sperm survival rates for most species remain around 50% (21). Notably, vitrification is simple to perform and does not require osmotic CPAs. Thawed sperm using this method exhibit significantly higher motility than those from slow freezing, and no significant differences in sperm DNA damage are observed, making vitrification a reliable alternative (22). Freeze-drying (lyophilization), a novel technique, enables the long-term maintenance of sperm DNA integrity at both low and room temperatures (23).

### 2.2 Methods and principles of oocyte cryopreservation

Oocytes are larger in volume, have a relatively smaller surface area, and contain high levels of intracellular fat granules compared to sperm and somatic cells. These characteristics make them more sensitive to low temperatures and the formation of intracellular ice crystals. Moreover, the zona pellucida and plasma membrane surrounding the oocyte impede the flow of cryoprotectants and water, thereby affecting cryopreservation efficiency. Oocyte cryopreservation methods thus differ significantly from those for sperm. Slow-freezing and vitrification are commonly used for oocytes. Notably, a lower cryoprotectant concentration reduces oocyte toxicity during slow freezing. The extended cooling time alleviates dehydration difficulties caused by the small surface-to-volume ratio of oocytes, ensuring adequate dehydration and minimal ice crystal formation (24). In 1977, Whittingham successfully achieved slow freezing of mature mouse oocytes, yielding blastocyst rates of 50%−70% in “*in vitro*” fertilized embryos post-thawing (4). Subsequent studies have increasingly focused on other factors, such as CPA types and removal methods (25, 26), and temperature (27, 28), and their influence on cryopreservation outcomes. Notably, slow freezing requires expensive programmable freezers and prolonged equilibration at low temperatures, making oocytes susceptible to physical and chemical damage that compromises thaw survival rates. As such, no optimal results have been achieved with oocyte cryopreservation by slow freezing.

Vitrification requires cryoprotectant concentrations of 6.0 mol/L or higher. Intracellular and extracellular fluids become highly concentrated during rapid cooling, leading to a sharp increase in viscosity and forming a non-crystalline solid (vitrification), thereby significantly reducing intracellular ice crystal formation (29, 30). In 1985, Rall et al. (31) successfully preserved mouse oocytes using vitrification, achieving a post-thaw survival rate of 87.8%. Subsequently, various methods, including the microtube, droplet, Cryotop, and open-pulled straw (OPS) methods, have emerged based on different carriers. The Cryotop and OPS methods are the most widely used. The OPS method was first applied in 1997 to cryopreserve 3–5-day-old bovine “*in vitro*” fertilized embryos. A small amount of cryoprotectant and embryos were siphoned into the straw, then rapidly immersed in liquid nitrogen, resulting in a blastocyst rate of 6.1% after thawing (32). Subsequently, this method was employed for the cryopreservation of bovine oocytes, achieving fertilization and blastocyst rates of 50% and 25%, respectively, upon thawing (6). The Cryotop method utilizes an extremely small volume (< 0.1 μl) of vitrification solution, enabling rapid passage of oocytes through the critical temperature zone. Oocytes cryopreserved using this method achieved a blastocyst development rate of 10.8% ± 1.4% after thawing and fertilization, which was significantly higher than that of the OPS method (5.3% ± 1.3%) (33). In summary, vitrification cryopreservation reduces cryoprotectant toxicity and cryoinjury compared to conventional methods. Its simplicity, time efficiency, and high success rate make it the optimal choice for oocyte cryopreservation today.

### 2.3 Methods and principles of embryo cryopreservation

Embryos and oocytes exhibit significant differences in ultrastructure and cell count. Some cells in embryos may be damaged or die during cryopreservation. Nonetheless, the remaining cells can potentially repair themselves and continue normal embryonic development. In contrast, oocytes exhibit lower tolerance to low temperatures and the chemical toxicity of cryoprotectants, making survival after damage challenging because they are single-celled structures. Oocyte cryopreservation methods are not entirely suitable for embryos. Common embryo cryopreservation techniques include slow freezing, rapid freezing, and vitrification.

In 1972, Whittingham et al. achieved cryopreservation of mouse embryos using slow-freezing techniques. Notably, thawed embryos transplanted into recipients produced normal offspring, thereby pioneering the field of embryo cryopreservation. The following year, slow freezing yielded the world's first calf born from frozen embryo transfer (3). A cryoprotectant mixture comprising 0.2–3.5 mol/L osmotic agents and 0.25–0.5 mol/L non-osmotic agents is employed when building upon slow-freezing techniques. The temperature is rapidly lowered to −35 °C to −30 °C after induced crystallization, and then the embryos are immersed in liquid nitrogen. This process reduces freezing time to under 2 h, thereby establishing a rapid-freezing method. Currently, this method is well-established, achieving over 90% normal morphology in thawed bovine embryos and over 55% pregnancy rates in recipient cows post-transfer. It has thus been widely applied for cryopreservation of embryos from other mammals, including cattle (6), sheep (8), and rabbits (7). It remains a commonly used method in modern livestock production (34).

Different vitrification methods yield varying preservation outcomes for embryos. Rail and Fahy (31) pioneered the use of the pipette technique for freezing mouse 8-cell embryos. This technique achieved a maximum post-thaw survival rate of 87.8%. Techniques, such as the two-step, OPS, and Cryotop method, have also been applied to embryo cryopreservation. These approaches exhibit improved cryopreservation protocols and a shift from “single” to “combination” cryoprotectants. The variety of cryoprotectants, including the incorporation of numerous low-toxicity biomimetic materials into cryopreservation, has also expanded significantly. These advancements substantially improve post-thaw embryo survival and implantation rates.

## 3 Freeze protection agents

CPAs are substances capable of protecting cells from damage caused by low or ultra-low temperatures (35). They shield cells during cryopreservation and thawing by dehydrating cells, adjusting osmotic pressure, reducing intracellular ice crystal formation, promoting vitrification, stabilizing intracellular proteins, and regulating extracellular electrolytes. CPAs achieve their ice-suppressing and cryopreservation-promoting functions through three mechanisms: inhibiting ice recrystallization, lowering the freezing point, and controlling ice morphology and ice crystal growth. Cryoprotectants are categorized into traditional and novel types based on characteristics, such as toxicity levels and biocompatibility.

### 3.1 Traditional antifreeze agents

Traditional CPAs are classified into permeable and impermeable types based on the selective permeability characteristics of cell membranes. The permeable CPAs include DMSO, GLY, 1, 2-propanediol, EG, and acetamide. These compounds possess relatively low molecular weights, which enables them to permeate cell membranes. They promote water efflux from cells to the extracellular space, thereby reducing intracellular ice crystal formation. They are thus also referred to as intracellular cryoprotectants. The mechanisms of action for various permeable CPAs are fundamentally similar. They bind water molecules via hydrogen bonds, reducing water activity by 40%−60% and lowering the solution's freezing point below −30 °C, thereby inhibiting ice nucleation (36). These CPAs also rapidly penetrate cell membranes to achieve intracellular-extracellular concentration equilibrium, thereby reducing cellular shrinkage rates caused by cryo-dehydration. Moreover, they stabilize the lipid bilayer structure of cell membranes, preventing membrane rupture by reducing fluidity at low temperatures. They also mitigate oxidative stress and protein denaturation, indirectly inhibiting ice crystal damage to biomolecules. However, permeable CPAs may exhibit toxicity at excessively high concentrations and potentially cause adverse effects on cellular genes, including exacerbating DNA fragmentation and reducing mRNA expression levels of multiple key genes (37–39). The toxicity and permeability of different CPAs vary significantly. In the same line, oocytes and embryos at different developmental stages exhibit varying sensitivities to different CPAs, leading to differing cryoprotection outcomes (1). For instance, cryotoxicity to mouse morula embryos increases in the order EG, GLY, DMSO, PG, and acetamide, with EG demonstrating the optimal cryoprotection effect (40). A vitrification medium composed of DMSO (20.5%), acetamide (15.5%), PG (10%), and polyethylene glycol (6%) used to preserve mouse embryos resulted in an 87.7% survival rate after thawing (19). In contrast, a cryoprotectant containing GLY (0.3 mol/L), 15% DMSO, and 15% EG used to vitrify bovine embryos via the OPS method achieved an 82.4% survival rate post-thawing (15). In another study, mouse oocytes treated with 1.5 mol/L DMSO and EG solutions at 23 °C for 15 min before cryopreservation exhibited significantly higher post-thaw survival rates than those treated with PG. However, combining low concentrations of DMSO and PG significantly reduced the toxicity of both cryoprotectants, thereby increasing cell survival (37). In a contrasting study, pig cumulus-oocyte complexes (COCs) were equilibrated in a 2% EG + 2% PG solution for 4–15 min before vitrification. The survival rate of thawed COCs was significantly higher than that of EG+DMSO (1:1) (73.8% vs. 51.1%) when the cryoprotective solution contained EG+PG (1:1). This phenomenon caused the total cell count of blastocysts after fertilization to be close to that of the unfrozen group (41).

Non-osmotic CPAs possess large molecular weights and cannot permeate cell membranes. They act solely in extracellular fluids, thereby reducing ice crystal formation. They are commonly referred to as extracellular cryoprotectants and include sucrose, trehalose (Tre), bovine serum albumin, fetal bovine serum, and fetal globulin. Among them, sugars constitute a major class of non-osmotic cryoprotectants. Compounds such as sucrose, trehalose, and raffinose have been extensively used for the cryoprotection of animal tissues (42, 43). Of note, they induce cellular dehydration by altering osmotic pressure during freezing. They prevent rapid water influx during thawing and expel cryoprotectants from cells, thereby reducing cellular damage. Sugars also function as osmotic buffers, slowing cellular swelling during thawing to minimize osmotic injury, consequently protecting cells. They form high-viscosity glassy matrices via hydrogen-bond networks, thereby elevating the glass transition temperature of extracellular solutions, inhibiting protein secondary structure changes, and enhancing cellular cryotolerance (44). For example, a 200 g/L sucrose solution exhibits a higher glass transition temperature (−30 °C) than equivalent concentrations of EG (−85 °C) and GLY (−65 °C) (45). Sucrose is thus employed in embryo and semen cryopreservation because it improves post-thaw survival, re-fertilization rates, and hatching rates of bovine *in vitro* fertilized blastocysts (46). It also enhances post-thaw motility in equine sperm, maintains cytoplasmic and acrosome membrane integrity, and significantly increases mitochondrial membrane potential (47). Trehalose exhibits lower reducing properties, thereby preventing non-specific interactions with biomolecules. Its hydrogen-bond network with water is denser and more stable than that of sucrose, thereby maintaining activity during freeze-thaw cycles and delivering superior cryoprotective effects compared to sucrose (48, 49). These attributes make it suitable for preserving cells with high freezing difficulty. For instance, 50 mM trehalose significantly increased the survival rate of mouse spermatogonial stem cells after thawing, i.e., after 1 week of freezing: 89.7% vs. 76.1% and after 3 months of freezing: 86.1% vs. 68.8% (50). In another study, replacing DMSO in the cryoprotectant with 200 mM trehalose resulted in a slightly higher survival rate of rat hepatocytes after thawing compared to the 5% DMSO group (80% vs. 75%). This change also significantly enhanced MTT metabolic activity, which demonstrated trehalose's potential as a standalone cryoprotectant (51). However, trehalose struggles to directly penetrate cell membranes. Multiple intracellular delivery systems have thus been developed to achieve the coexistence of trehalose in both intracellular and extracellular fluids. These delivery systems enhance cellular freeze tolerance and lay the groundwork for trehalose to replace permeable CPAs. For instance, colloidal nanoparticles encapsulating trehalose using aminoethyl phosphate and hexametaphosphate achieve efficient delivery, attaining intracellular concentrations as high as 237 ± 8.54 mM in human smooth muscle cells with a loading efficiency of 137.3 ± 34.5%. The cell survival rates increased by 82.3 ± 3.6% following freeze-thaw cycles, and exhibited no significant difference compared to the DMSO control group (81.3 ± 5.6%) (52).

Though traditional CPAs effectively inhibit ice crystal formation by lowering the freezing point of solutions, their high concentrations cause toxic damage to cells, leading to reduced cell viability and impaired embryonic development. Identifying and developing novel low-toxicity CPAs for tissue and cell cryopreservation is thus a research priority.

### 3.2 New types of freeze protection agent

**Figure 1:**
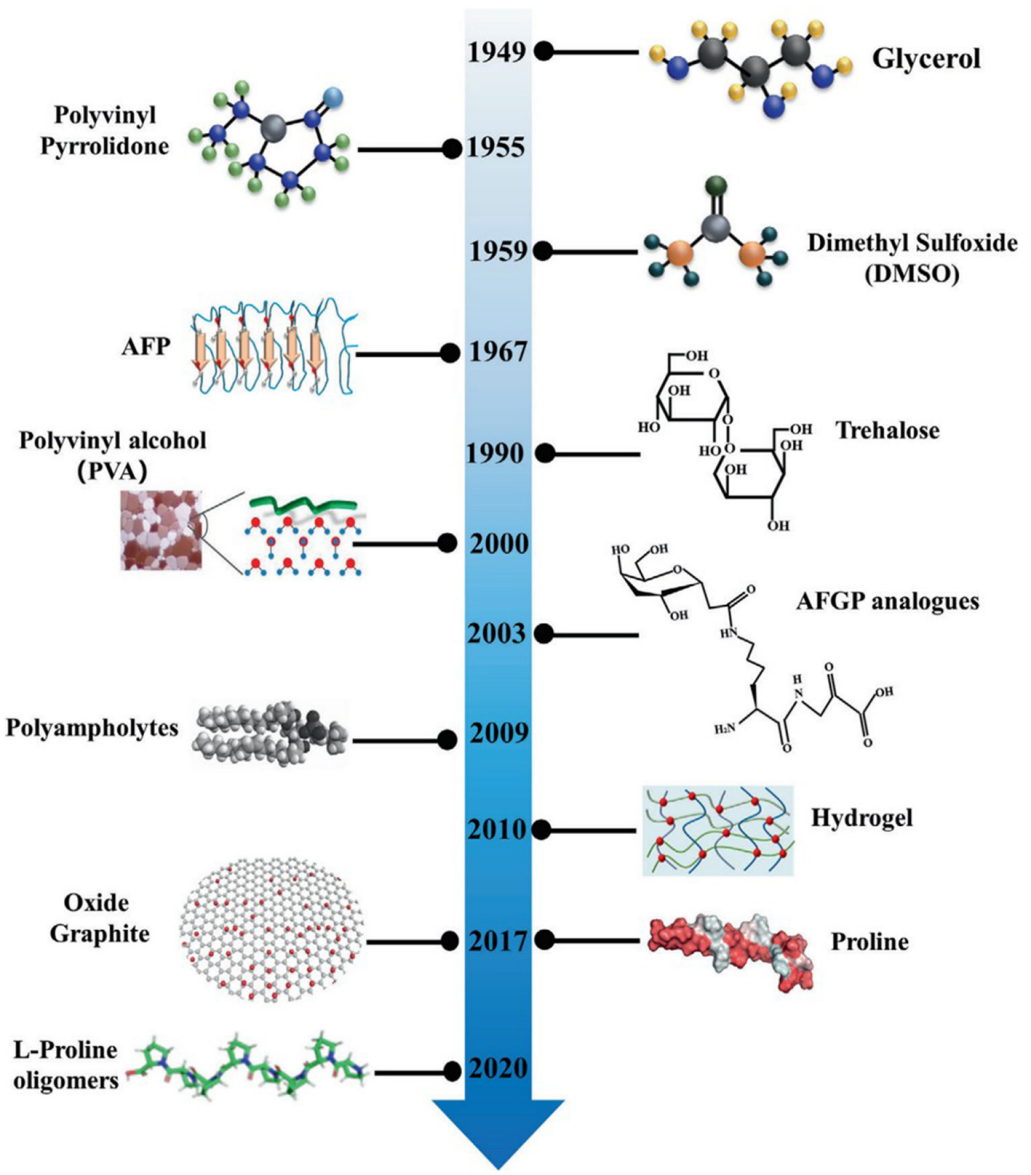
Milestone events in modern cryobiology and nanomaterials-assisted cell cryopreservation. Adapted from (53), licensed under CC BY 4.0; and includes elements reproduced with permission from American Chemical Society (Copyright 2013, 2016, 2020) and from Wiley-VCH (Copyright 2017).

The rapid advancement and interdisciplinary convergence of biochemistry and materials science have led to precise control over ice crystal nucleation and growth. These solutions are products of innovative technologies based on biomimetic material design and biomolecular modification, which provide breakthroughs in the cryopreservation of cells and tissues (Figure 1) (53). Novel CPAs with excellent biocompatibility and low toxicity can replace toxic reagents like DMSO. These CPAs can effectively mitigate mechanical damage and osmotic shock during cryopreservation, thereby enhancing post-thaw cell survival and function by inhibiting ice crystal growth and recrystallization. These attributes demonstrate their broad application potential as next-generation cryoprotectants. Currently, novel CPAs primarily include AFPs, nanomaterials, synthetic polymers, polysaccharides, and eutectic solvents. The key characteristics of traditional and novel biomimetic cryoprotectants were summarized in Table 1, ice inhibition mechanisms, typical concentrations, main advantages, limitations, and common applications. This comparative overview provides a foundation for understanding the distinctions between conventional CPAs and emerging biomimetic alternatives.

**Table 1:** Comparison of characteristics between traditional and novel biomimetic cryoprotectants.

CPA Category	Subtypes/Representatives	Membrane permeability	Ice inhibition mechanism	Typical concentration	Main advantages	Main limitations	Common cell types/species	References
Traditional CPA	DMSO, Glycerol, EG, PROH	Permeable	Binding water molecules via hydrogen bonds, lowering freezing point; increasing proportion of unfrozen water; stabilizing lipid bilayer structure of cell membrane	1.0–2.0 mol/L (slow freezing); 4–6 mol/L (vitrification); 5–15% (v/v)	High permeability; strong vitrification ability; mature clinical application	Cytotoxicity; DNA methylation alterations; histone modifications; affecting gene expression; difficult clinical removal	Stem cells, oocytes, embryos, sperm	(3, 29, 37–39, 109, 110)
	Sucrose, Trehalose, Raffinose	Non-permeable	Hydration; regulating ice crystal morphology; IRI activity; forming glassy matrix; serving as osmotic buffer	0.1–0.5 mol/L	High biocompatibility; non-toxic; natural source; high glass transition temperature	Unable to penetrate cell membrane; requires auxiliary delivery system	Sperm, red blood cells, stem cells, hepatocytes	(44, 45, 48–51)
	PVP, HES, PEG	Non-permeable	Steric hindrance effect; inhibiting ice crystal growth; stabilizing extracellular environment	5%−20% (w/v)	Macromolecular stabilization; extracellular protection	IRI activity lower than PVA; efficiency dependent on molecular weight	Red blood cells, embryos, stem cells	(111–113)
Novel Biomimetic CPA	Antifreeze proteins (AFPs)	Variable	Adsorption-inhibition mechanism; thermal hysteresis (TH); dynamic ice shaping (DIS); IRI activity; Janus effect dual regulation of ice nucleation	0.1–10 mg/ml or 1–500 ng/ml	Natural ice inhibition mechanism; multifunctional (TH, DIS, IRI); effective at extremely low concentrations; protects cell membrane structure	Risk of needle-like ice crystals; immunogenicity; high cost; toxicity at excessive concentrations; species specificity	Oocytes, sperm, embryos, ovarian tissue	(114–119)
	Synthetic polymers (PVA, polyproline, polyampholytes)	Non-permeable	Hydroxyl spacing matches ice crystal lattice (2.92 Å vs. 2.74 Å); disrupting quasi-liquid layer; amphiphilic structure; charge balance	0.1–10 mg/ml or 0.05–50 mM	Controllable structure; high stability; non-immunogenic; high IRI activity; strong designability	Polymerization degree affects activity; biodegradability differences	Red blood cells, stem cells, oocytes, fibroblasts	(10, 86, 88, 89, 112, 113)
	Nanomaterials (GO, OQCNs, MOF, Fe3O4)	Non-permeable	Lattice matching (7.42 Å matching ice crystal); decisive role of hydroxyl density; hydrogen bond network regulation; photothermal/magnetothermal conversion achieving rapid uniform rewarming	0.001–0.1 wt% or 0.5–10 mg/ml	Multifunctional (ice inhibition + rapid rewarming); extremely low concentration; surface modifiable; enables uniform rewarming of large-volume samples	Long-term toxicity unknown; high distribution uniformity requirement; difficult *in vivo* clearance; requires external physical field	Sperm, red blood cells, oocytes, stem cells, fibroblasts, kidney, heart	(70, 75, 120–123)
	Hydrogels (Alginate)	Non-permeable	Restricting free water; bound water antifreeze; physical barrier inhibiting ice crystal propagation; 3D network structure stabilizing cells	0.5%−2% (w/v)	3D biomimetic environment; reducing CPA concentration; easy removal; providing extracellular matrix-like environment	Mass transfer limitations; requires encapsulation/recovery steps	Stem cells, embryos, β-cells, tissue engineering, islet cells	(124–127)
	Polyelectrolytes/Zwitterionic molecules (Betaine, L-Carnitine)	Non-permeable	Charge balance disrupting interfacial water molecules; stabilizing cell membrane; inhibiting ice nucleation and growth	1–2 mol/L	High biocompatibility; stabilizes cell membrane; inhibits ice nucleation; partially naturally available	Insufficient mechanism research; some require combination with other CPAs	Sperm, red blood cells, chondrocytes, stem cells	(86, 89, 128, 129)

#### 3.2.1 AFPs

**Figure 2:**
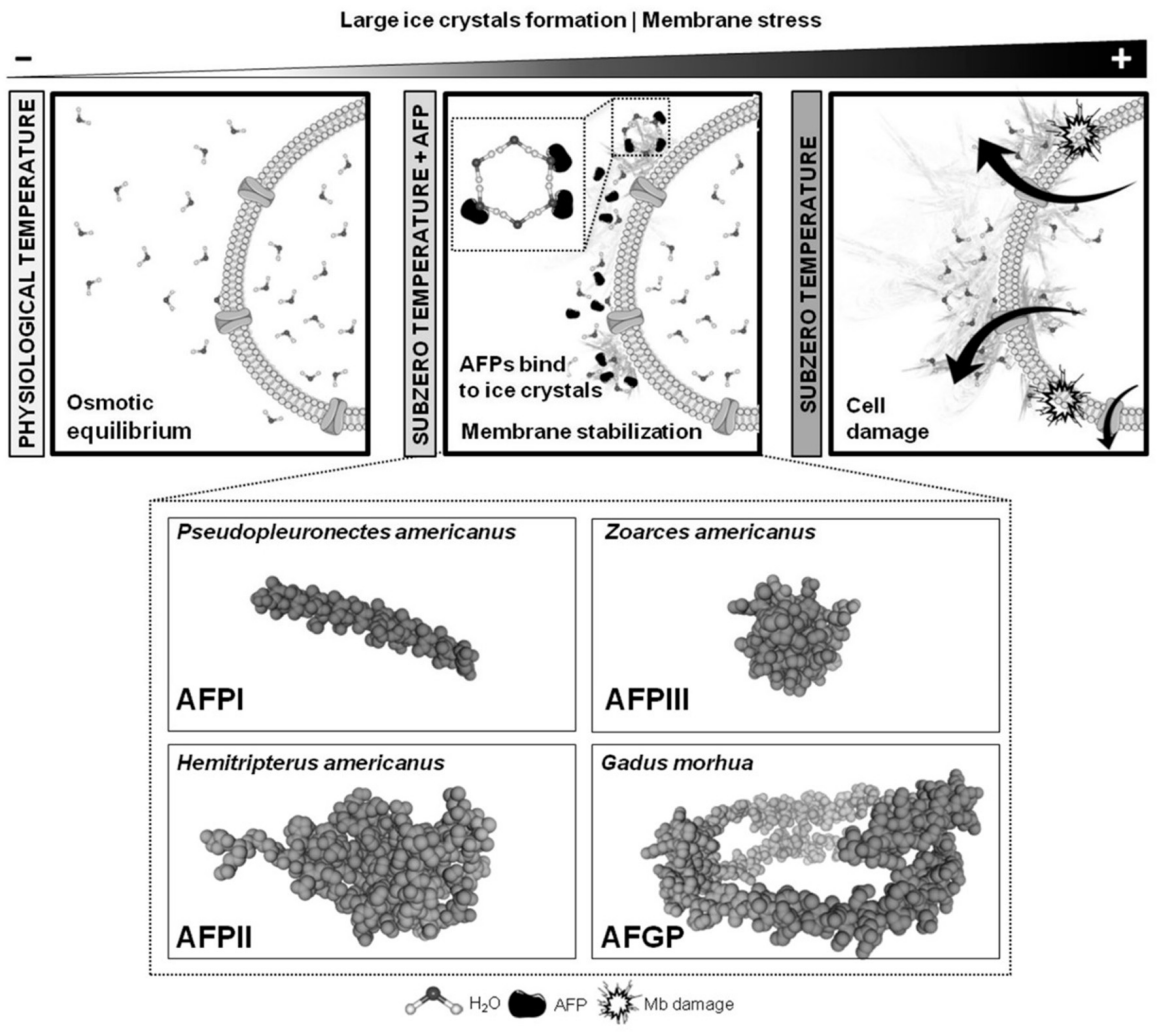
Antifreeze protein (AFP) mechanisms of the action binding to forming ice crystals around gametes or the embryo, and 3D models of the most used AFPs in reproductive technologies. The 3D models were created using the SWISS-MODEL online platform with the following National Center for Biotechnology Information (NCBI) sequences: AFPI (Pseudopleuronectes americanus; GeneBank ID: AAA49466.1), AFPII (Hemitripterus americanus, GenBank ID: AAA49618.1), AFPIII (Zoarces americanus; GenBank ID: ABA41371.1), and AFGP (Gadus morhua; GenBank ID: AAQ09567.1). Adapted from (57), licensed under CC BY 4.0.

Organisms have evolved multi-layered survival mechanisms through physiological regulation, metabolic pathway restructuring, and gene expression control to adapt to extreme sub-freezing temperatures. The synthesis and secretion of AFPs represent a crucial molecular manifestation of this evolutionary strategy. The first AFPs were discovered and reported in polar marine fish in the 1960s. They demonstrated their ability to significantly mitigate cold-induced freezing damage (54). To date, natural AFPs have been identified in diverse organisms, including fish, insects, and bacteria (55, 56). Notably, they have become a research hotspot in veterinary cryobiology due to their unique capabilities to regulate ice crystals (Figure 2) (57).

The core mechanisms of AFPs' low-temperature antifreeze activity are thermal hysteresis (TH) and ice recrystallization inhibition (IRI). The IBF of antifreeze proteins interacts with ice crystal surfaces at low temperatures, thereby non-additively lowering the temperature at which ice crystal growth occurs, i.e., the solution freezing point, while having a minimal effect on the melting point temperature. This phenomenon creates a difference between the melting and freezing points of ice crystals, resulting in the thermal hysteresis (58). Ice recrystallization typically occurs during the thawing process of cryopreserved samples. Small ice crystals gradually dissolve within the existing ice crystal system, while large ice crystals continuously grow in size, exerting mechanical compression and damage on cells or tissues. Their IBF competitively adsorbs onto ice crystal surfaces in the presence of AFPs via specific interactions, such as lattice matching and hydrogen-bond networks, thereby occupying growth sites for both small and large crystals. This phenomenon prevents the directed migration of water molecules toward the surfaces of large crystals, thereby reducing the growth substrates for large crystals (59). In addition, the steric hindrance effect of AFP molecules physically impedes the fusion of small and large ice crystals, thereby inhibiting recrystallization (58, 60). AFPs alter the thermodynamic equilibrium of the ice crystal system through interfacial adsorption, with the core mechanism being the amplification of the Gibbs-Thomson effect. The originally ordered water molecules are replaced with more disordered protein-water complexes when AFPs bind to ice crystals, significantly increasing the system's entropy. Moreover, high-enthalpy interactions between the protein surface and water, such as hydrogen bonds between polar residues and water molecules, create an energy barrier that hinders further crystal aggregation and growth. This synergistic entropy increase-enthalpy barrier effect elevates the recrystallization barrier, trapping the ice crystal system in a metastable state. The metastable state maintains the fine, dispersed geometric morphology of ice crystals. This energy barrier effect constitutes one of the primary mechanisms by which antifreeze proteins resist cold-induced damage (61).

Natural AFPs have been widely used for cryopreservation of sperm, oocytes, and embryos. For instance, adding 1 μg/ml AFP III to the dilution medium significantly improves goat sperm motility, membrane integrity, acrosome integrity, and mitochondrial function (62). For instance, adding 1 μg/ml AFP III to the dilution medium significantly improves goat sperm motility, membrane integrity, acrosome integrity, and mitochondrial function (62). It also significantly increases the proportion of rapidly motile sperm in thawed frozen Japanese white rabbit semen (63). In another study, the addition of 40 mg/mL antifreeze glycopeptides (AFGPs) during vitrification of mouse and pig oocytes significantly increased post-thaw survival rates to 82% and 25%, respectively, compared to 0% in the control group. AFGPs also significantly enhanced the structural integrity of oocyte membranes (64). Adding 500 ng/ml AFP III to the vitrification solution effectively protected the integrity of cell membranes, spindles, and chromosome structures, thereby significantly improving mouse oocyte thaw survival rate (94.6% vs. 84.5%) and blastocyst formation rate (89.1% vs. 68.9%) (65). Of note, similar results were obtained with AFGP8 in the vitrification of bovine oocytes (66). Arctic yeast-derived LeIBP exhibits significant protective effects on vitrified bovine embryos. It enhances embryo cryotolerance and effectively improves post-freezing developmental capacity through various mechanisms, such as reducing apoptosis rates and maintaining total cell numbers, thereby demonstrating overall superior performance compared to AFP III (67). Pre-warming bovine embryos stored at low temperature (4 °C) for 60 min at 37 °C in an AFP-containing medium enables embryo survival for up to 10 days, whereas non-pre-warmed embryos survive only up to 7 days (68). It is therefore evident that, despite the cryoprotective functions of AFPs, including TH, IRI, and membrane protection, their efficacy is influenced by multiple factors, such as species, cell type, or embryonic developmental stage, concentration, and cryopreservation protocol. Current CPAs still possess numerous limitations, and marked differences exist in the suitability and conditions of AFPs for cryopreserving livestock gametes and embryos. Furthermore, strategies for combining AFPs with other agents to mitigate CPA toxicity, as well as the long-term effects of AFPs, remain to be elucidated. Future studies should therefore focus on these areas to facilitate the incorporation of AFPs into improved CPA formulations, thereby rendering them safer, more efficient, and more practical.

#### 3.2.2 Nanomaterials

**Figure 3:**
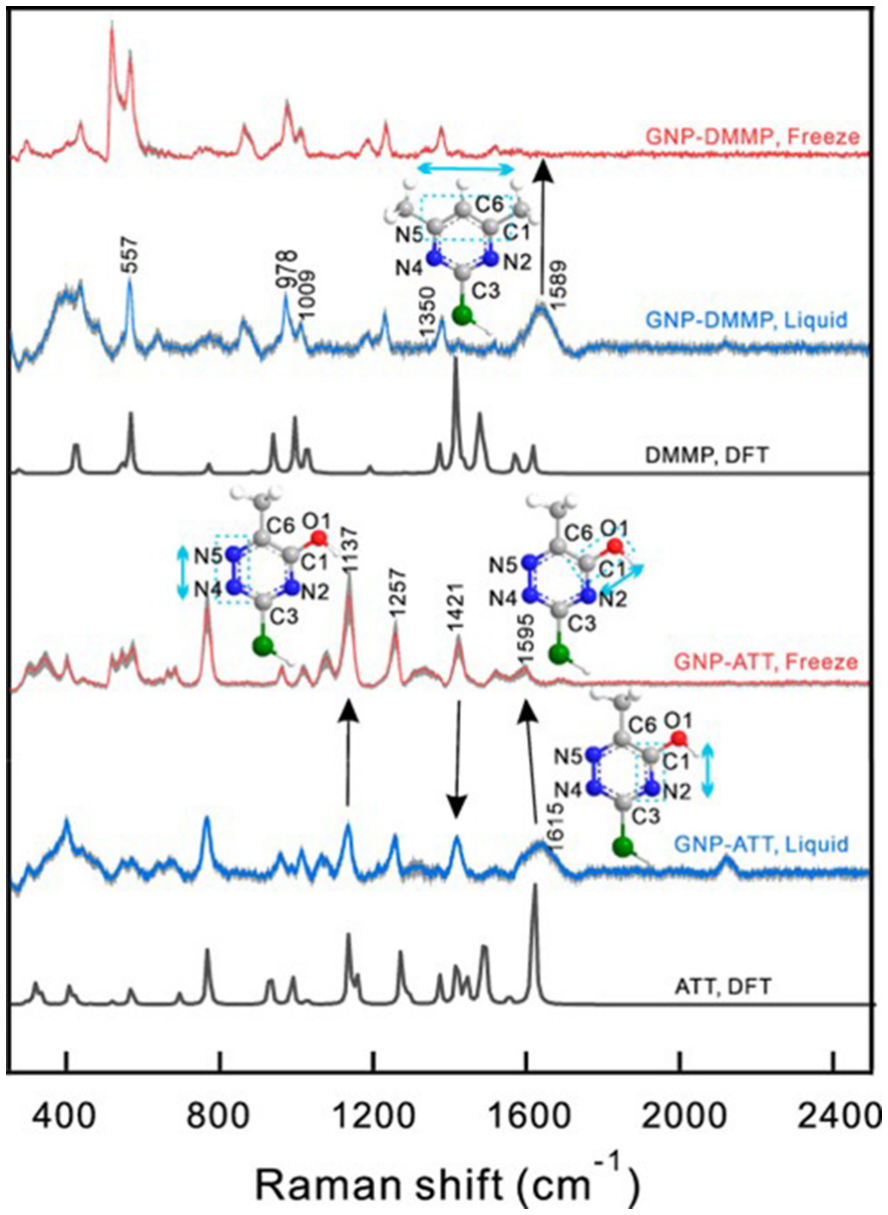
SERS spectra of GNP-ATT and GNP-DMMP in liquid sols (blue lines) and frozen ices at −20 °C (red lines), respectively. DFT-calculated Raman spectra of pure ATT and DMMP in liquid are given as references (black lines) (69). Adapted from (69), with permission from American Chemical Society (Copyright 2022).

The core mechanism of nanomaterials' freeze resistance lies in IRI. IRI mimics adsorption-inhibition processes by surface chemical modification, alters the curvature of the ice-water interface, and suppresses ice crystal growth. However, this mechanism relies more on the synergistic effects of hydroxyl density and rigid surfaces. The construction of a monolayer model of small molecules on a gold nanoparticle surface and surface-enhanced Raman spectroscopy analysis revealed that the gold nanoparticle-6-aza-2-thiothymine conjugate (GNP-ATT) compound exhibits IRI activity (Figure 3) (69). This phenomenon indicates that its hydroxyl groups form hydrogen bonds with ice crystals. A higher hydroxyl density enhances the hydrogen bonding capacity between ice crystals and the small molecule, leading to more pronounced IRI effects. Hydroxyl density is thus a decisive factor influencing IRI activity. In contrast, hydrophobic groups, such as methyl, slightly hinder hydrogen bond formation. In addition, the rigid surface of gold nanoparticles imposes geometric constraints, enabling a highly matched lattice spacing between hydroxyl groups and ice crystal edges, thereby significantly enhancing binding efficiency. This “templating effect” resembles the ice-binding surface structure of natural AFPs and exhibits high similarity in molecular recognition mechanisms and functional design (69).

Certain nanomaterials, such as graphene oxide (GO), also exhibit structures similar to those of AFPs. The hydroxyl groups on their surfaces can form hydrogen bonds with water molecules, leading to preferential adsorption of ice crystals onto the GO surface and curved ice crystal surfaces. This phenomenon lowers the freezing temperature and inhibits ice crystal growth (70). For instance, adding just 0.01% GO to culture medium significantly enhances the viability of frozen-thawed horse sperm (71). Various types of oxidized quasi-carbon nitride quantum dots (OQCNs) also exhibit TH and IRI activity. In these activities, the density of effective adsorption sites is a crucial factor determining their ice crystal growth inhibition. In a previous study, applying OQCNs to sheep red blood cell cryopreservation significantly improved recovery survival rates, reaching 2.2 times those of commercial hydroxyethyl starch (70). Notably, cryopreservation of red blood cells using trehalose encapsulated with various nanomaterials has yielded favorable outcomes. For instance, delivery of trehalose to sheep red blood cells using the graft copolymer PP-50 increased post-thaw survival by 20.4% ± 5.6%, while co-incubation with 100 μg/ml PP-50 and 360 mM trehalose achieved intracellular trehalose concentrations of 123 ± 16 mM and uptake rates of 34.2% (72). Trehalose efficiently permeated cells via translocation through the lipid bilayer when apatite served as the carrier, achieving a thaw survival rate of 90.7%, which was approximately 40% higher than with trehalose alone. In culture medium containing apatite nanoparticles, the hemolysis rate of red blood cells remained below 8%. Of note, apatite nanoparticles induce only transient membrane permeability changes lasting approximately 5–15 min unlike other nanomaterials. The carrier does not invade the cell after trehalose molecules complete transmembrane transport (73). This mechanism of action is mild and reversible, thereby preserving cell membrane integrity and ensuring high safety.

The deepening of research on organic cryoprotectants has led to increased attention to the IRI activity of inorganic materials. Inorganic salts possess inherent advantages in low-temperature applications, such as certain salts (e.g., NaCl and CaCl_2_) that lower melting points by disrupting the hydrogen-bond networks of water molecules. Moreover, selenium nanoparticles regulate ice crystal growth kinetics, with selenium atoms on the surface exhibiting specific interactions at the ice crystal interface that significantly inhibit ice recrystallization. Adding selenium nanoparticles to diluents reduces apoptosis and membrane lipid peroxidation during bovine sperm cryopreservation, thereby improving post-thaw sperm motility and *in vivo* fertilization rates (74). The GO-Fe3O4 nanocomposite, formed by coupling GO with magnetite (Fe3O4), achieves synergistic photo-magnetic dual responsiveness. Notably, it rapidly and uniformly generates heat, with a heating rate up to 5 °C/s, under external magnetic field and near-infrared-light stimulation. The oxidized groups on the GO surface also regulate ice nucleation through a hydrogen bond network, controlling crystal size below 20 μm (one-fifth that of conventional ice crystals) and inhibiting ice recrystallization, thus enhancing cryopreservation efficacy. In a previous study, cryopreservation of mesenchymal stem cells using GO-Fe3O4/alginate hydrogel composites resulted in cell viability and proliferation at days 0, 3, and 7 post-cryopreservation that matched those of non-cryopreserved cells, demonstrating the nanocomposite's effective preservation of MSC activity. Furthermore, the simple preparation process and controllable cost of this material render it more suitable for large-scale application in livestock (75). The zirconium-oxygen cluster structure of zirconium acetate exhibits ice-crystal-regulation properties similar to those of AFPs, promoting ice crystal growth along the c-axis and thereby forming hexagonal prismatic structures. This phenomenon significantly reduces ice crystal growth rates and demonstrates strong IRI activity (76). Nanomaterials have demonstrated significant advantages in cryopreservation. Breakthroughs in intelligent design of nanocomposites (e.g., thermosensitive nanogels) and atomic-level manufacturing technologies would advance cellular cryopreservation toward higher viability maintenance and lower damage rates, providing reliable assurance for clinical-grade cell therapies.

#### 3.2.3 Synthetic polymers

Synthetic polymers are functional, biomimetic materials for AFPs that demonstrate immense application potential due to their excellent biocompatibility, low toxicity, and synthetic accessibility. These materials encompass small molecules, macromolecules, and amphiphilic polymer zwitterionic electrolytes. Their antifreeze mechanism operates as a multiscale, multi-mechanism synergistic regulatory system, offering novel insights for low-temperature biology and bioengineering applications. The antifreeze mechanism of synthetic polymers precisely regulates ice crystal formation and growth through multidimensional interactions at ice crystal interfaces, dynamic hydrogen-bond network formation, and steric hindrance. Eniade et al. (77) first reported that small-molecule carbohydrates mimicking AFGPs also exhibit IRI activity as ice crystal inhibitors, with their active carbohydrate groups contributing to IRI capability. Small-molecule AFGP analogs disrupt three-dimensional hydrogen bonds in bulk water, thereby reducing the drivers of ice crystal growth. They also interfere with water molecule transfer pathways to the quasi-liquid layer, thereby inhibiting ice recrystallization (78). The novel small-molecule compound N-aryl-d-alanyl amide mitigates cryoinjury in hematopoietic progenitor cells, thereby increasing post-freezing cell recovery rates to 2.4 times the original level (79).

Macromolecular polymers have even broader applications. The inter-hydroxyl spacing of 2.92 Å in polyvinyl alcohol (PVA) molecular chains closely matches the hydroxyl spacings (2.76 Å and 2.74 Å) on the primary and secondary prismatic faces of ice crystals. This spatial structural alignment enables PVA to target binding to the prismatic faces of ice crystals. Hydroxyl groups form hydrogen bonds with the ice crystal surface, creating a physical barrier that inhibits ice crystal expansion and recrystallization (80). Notably, PVA's antifreeze performance depends on its degree of polymerization. Optimal antifreeze effects are observed between 15 and 20 (81, 82). Irregular PVA structures exhibit superior diffusion on ice surfaces and a stronger affinity for both ice and water surfaces, thereby enhancing IRI activity. Combining PVA with biomimetic block copolymer worms significantly enhances IRI activity, achieving a 68% red blood cell survival rate post-resuscitation. This survival rate approaches that achieved with commercial cryopreservation protocols (70% survival with hydroxyethyl starch-HES + PVA) and is achieved with lower use of a toxic organic solvent (83). Synthetic polyproline possesses the same type II helical structure as AFPs, and thus significantly inhibits ice recrystallization. Moreover, the electrostatic repulsion between charges in amphiphilic molecules effectively suppresses ice crystal growth (84). In a previous study, the average maximum ice crystal grain sizes when using L-proline oligomers (L-Pro, L-Pro3, L-Pro8, and L-Pro15), EG, and DMSO as CPAs were 42, 52, 25, 40, 55, and 70 μm, respectively, indicating that L-Pro8 provided the optimal anti-freezing effect. Adding 50 mM L-Pro8 (monomer concentration 0.4 M) to the vitrification media increased the survival of mouse oocytes during cryopreservation to 99.11%. The oocytes had significantly elevated mitochondrial membrane potential and ATP levels, indicating markedly improved mitochondrial function (10).

Polyampholyte polymers consist of both positively charged functional groups (e.g., amino groups) and negatively charged functional groups (e.g., carboxyl and sulfonic acid groups). These polymers exhibit overall electrical neutrality at specific pH levels. Locally present dipole structures enable them to form strong hydrogen-bond networks with water molecules, thereby interfering with ice crystal nucleation and growth. In addition, polyampholytic electrolyte molecules attach to cell membranes, thereby maintaining membrane structural stability. This effect increases with polymer concentration and the introduction of hydrophobic groups (85). Carboxylated polylysine (COOH-PLL), which exhibits low cytotoxicity and high cryopreservation efficiency, was the first polyampholyte used for cryopreservation (86). It demonstrates good compatibility with various cell types and has successfully enabled cryopreservation of multiple cell lines. A previous study demonstrated that COOH-PLL can replace DMSO for vitrification of mouse pronuclear-stage embryos up to three times, with embryos retaining high developmental capacity (61.2% ± 3.1% vs. 44.2% ± 2.7%), comparable to that of fresh embryos (70.0% ± 3.6%) (87). In the same line, there was no significant hemolysis when sheep red blood cells were cryopreserved with polyampholyte concentrations up to 40 mg/mL, indicating their compatibility with red blood cells (88). Methacrylate copolymers possess both random positive and negative charges (with a net charge tending toward zero), which enables them to interact with the outer surface of cell membranes. This interaction protects membranes from various cryo-induced damages, facilitating the cryopreservation of mouse fibroblasts, human fibrosarcoma cells, and human adipose-derived stem cells, with a survival rate of >90% (89). Methylethylene ether-maleic anhydride polymers increased the cryopreservation survival rates of human lung cancer cells to 88%. Given the dual mechanisms of ice inhibition and membrane stabilization exhibited by synthetic polymers in various mammalian cells and embryos (90, 91), their successful application provides new research perspectives for the efficient cryopreservation of livestock gametes and somatic cells. In particular, they demonstrate significant application potential in reducing cryoprotectant toxicity, enhancing embryonic developmental capacity, and preserving sperm membrane integrity.

#### 3.2.4 Polysaccharides

Antifreeze polysaccharides are highly promising biomimetic antifreeze materials that function through precise regulation of ice crystal growth at the molecular level. Their efficient interactions with water molecules through hydrogen bond networks and stable protection of biological membrane structures form a multidimensional synergistic antifreeze system. Polysaccharides are also natural biomacromolecules that possess both biocompatibility and antioxidant activity, offering a green, low-toxicity, and highly effective solution for cryopreservation (92). Research on antifreeze polysaccharides began relatively late, with the first successful extraction of ice-nucleating polysaccharides containing polyacetyl-D-glucosamine groups and demonstrating high IRI activity occurring in 2002 from *Bacillus thuringiensis*. Notably, the supercooling depth decreased by 7.2 °C (from −5.9 °C to −13.1 °C) when this polymer was used to cryopreserve chicken livers, confirming its cryoprotective potential for the first time (93). The cuticle layers of the Alaskan beetle and mustard leaves contain mannose xylans with similar structures. However, the cuticle layers of the Alaskan beetle exhibit HTTH (up to 3.7 °C) and IRI activity while maintaining cell membrane stability, thereby enhancing cellular frost resistance (94). In contrast, the cuticle layers of mustard leaves primarily exert their antifreeze effect by inhibiting ice crystal growth, yet exhibit higher IRI activity. Arabinoxylan (AX) in wheat bran forms a complex network through the synergistic interaction of β-(1, 4)-D-xylose backbone and α-L-arabinose side chains. Its ice crystal growth inhibition rate ranges from 15% to 72% at −8 °C. Of note, AX with low branching and appropriate molecular weight exhibits superior ice crystal regulation capacity and lower ice crystal growth rates (95).

Advancements in research on anti-freezing mechanisms have led to the use of various polysaccharides for the cryopreservation of gametes. For instance, the addition of epimedium polysaccharide to the cryopreservation dilution solution of human semen resulted in significantly reduced levels of malondialdehyde and reactive oxygen species in thawed sperm. The treated group exhibited significantly higher sperm survival rates, higher normality rates of head morphology, and greater integrity of both the plasma membrane and the acrosome membrane compared to the untreated group (96). In another study, adding carboxymethyl chitosan to semen cryoprotective diluents improved mitochondrial membrane, plasma membrane, and acrosome integrity in boar sperm, effectively enhancing post-thaw sperm motility. Optimal effects were observed at a concentration of 0.6 mg/L (97). Adding exopolysaccharide ID1 (EPS ID1) to bovine embryo vitrification solution significantly increased post-thaw blastocyst re-cavitation and hatching rates, restored BAX gene expression to pre-freezing levels, and significantly lowered the rates of frozen embryos with abnormal blastocyst development compared to embryos without ID1 supplementation (98). Adding 400 mg/L Lycium polysaccharide to the cryoprotectant solution for rats ovarian tissue cryopreservation effectively maintained post-transplant ultrastructure and significantly improved follicle survival (99). Chemically modified chondroitin polysaccharides formed by incorporating threonine also increase the number of hydrogen bond donors. This modification reduces the average maximum ice crystal grain size from 70 to 25 μm and significantly enhances IRI activity, further advancing and expanding polysaccharide applications in cryopreservation (100).

#### 3.2.5 Deep eutectic solvent

In recent years, deep eutectic solvents (DES) have attracted significant attention due to their high biodegradability, low cost, high efficiency, and low toxicity. DES can be composed of natural primary metabolites, such as sugars, amino acids, organic acids, or choline derivatives, and are thus referred to as natural deep eutectic solvents (NADES). Mixing these components in specific molar ratios results in a solvent system that exhibits a melting point lower than that of its individual components and remains liquid at or near room temperature. DES were first reported in the early 21st century, with early research primarily focusing on systems composed of choline chloride and hydrogen bond donors, such as carboxylic acids, urea, succinic acid, and glycerol (101, 102). Subsequent studies shifted emphasis to NADES (Table 2) that naturally occur in animals and plants. Trehalose, glucose, sorbitol, and proline are substances produced by organisms in frigid environments to withstand low temperatures. These substances thus play crucial roles in winter metabolic pathways (103). For instance, urea and glucose levels increase significantly in wild tree frogs in November, when ambient temperatures drop to approximately −20 °C. These compounds slow cellular contraction during extracellular water crystallization while reducing tissue water content upon entering cells, thereby minimizing ice crystal damage. Metabolites, such as urea and glucose, thus form NADES through intermolecular interactions, a core mechanism that enables tree frogs to withstand extreme cold (104). NADES are a novel class of green solvents that exhibit their most distinctive feature in unique non-additive physicochemical behavior. This property originates from the coordination interactions between hydrogen bond donors and acceptors, which form stable hydrogen bond networks among two or more components, thereby constructing specific supramolecular systems (105, 106). Notably, the molar ratio of components is critical for liquid NADES systems.

**Table 2:** NADES identified in extremely cold-tolerant animals.

Component 1	Component 2	Component 3	Ratio	Ref.
Proline	Sorbitol	–	1:1	(101)
Proline	Glucose	–	5:3	(101)
Proline	Glucose	–	1:1	(101)
Serine	Glucose	–	5:4	(101)
Glutamic salt	Glucose	–	1:1	(101)
Glucose	Frutose	–	1:1	(105)
Urea	Sorbitol	NH_4_Cl	2:7:1	(130)
Urea	Glucose	CaCl_2_	4:5:1	(131)
Urea	Glucose	CaCl_2_	3:6:1	(131)

For instance, NADES composed of trehalose and glycerol (Tre: Gly = 1:30) lowers the freezing point and effectively inhibits ice crystal formation. Of note, its toxicity to mouse fibroblasts is significantly lower than that of DMSO. In a previous study, the cell survival rate and proliferation capacity of the NADES-treated group did not differ significantly from those of the DMSO group after 3 months of cryopreservation (107). Two NADES formulations with different compositions and molar ratios (glucose:urea: proline = 1:1:1 and proline: glucose = 5:3) significantly lowered the ice crystallization temperatures. Complete inhibition of ice crystallization occurred when the first formulation contained ≥70% NADES and the second formulation contained ≥80% NADES, which maintained the system in a glassy state. NADES exhibited lower toxicity to mouse fibroblasts compared to DMSO, with no significant difference in cell viability after thawing (108). Notably, NADES prepared with a 5:3 molar ratio of proline to glucose reduced the crystallization and melting temperatures to −7.1 °C and −15.1 °C, respectively. Further addition of 50% proline-glucose (5:3) lowered the crystallization temperature to −35.9 °C, with no crystallization observed above −50 °C (136). In summary, leveraging their tunable hydrogen-bond networks and superior ice-inhibition capacity, NADES offer a novel green medium for the cryopreservation of livestock gametes and embryos, while significantly reducing cytotoxicity. This solvent is expected to play a key role in mitigating osmotic injury and enhancing post-thaw developmental potential, thereby promoting the advancement of livestock germplasm resource conservation and assisted reproductive technologies toward greater efficiency and safety.

## 4 Conclusions and future prospects

Cryopreservation at ultra-low temperatures is a core technology for the long-term, stable preservation of animal germplasm resources, including gametes, embryos, and somatic cells, which are crucial for livestock propagation and population improvement. The representative studies of novel biomimetic cryoprotectants in gamete and embryo cryopreservation across different species were summarized in Table 3. The summarized outcomes, including post-thaw survival rates, motility parameters, blastocyst development, and functional assessments, highlight the potential of these emerging agents in reproductive cryobiology. Biomimetic CPAs such as nanomaterials, synthetic polymers, and polysaccharides have been successively developed owing to the deepening understanding of the structure and function of AFPs. These agents significantly enhance the post-thaw viability of gametes and embryos, reduce the cytotoxicity of traditional cryoprotectants, and improve cryopreservation efficiency by mimicking the structures and ice-crystal-regulation mechanisms of antifreeze proteins. Notably, the use of non-permeable CPAs delivered via nanotechnology to achieve dual inhibition of intracellular and extracellular ice crystals holds broad application prospects. Despite the significant progress in bioinspired CPAs, their mechanisms remain unclear. Controversy persists regarding the relationship between protectant structure and the regulation of ice crystal formation, growth, and morphology. Quantitative methods for assessing ice inhibition are yet to be established. Future studies should therefore focus on deciphering the structural and functional similarities between different AFPs and bioinspired CPAs to elucidate their antifreeze mechanisms. Concurrently, controversy persists regarding the degree of ice inhibition achieved by different materials, and quantitative analytical methods for assessing this inhibition remain to be established. Therefore, systematic evaluation of the specific effects of novel biomimetic materials on livestock gametes is required, alongside the development of a more reliable toxicity assessment system. Such efforts are essential to facilitate their translational application in the field of animal reproduction.

**Table 3:** Representative studies of novel biomimetic cryoprotectants in gamete and embryo cryopreservation.

Preservation target	Species	Cryoprotectant	Concentration	Outcome	References
Sperm	Goat	AFP III	1 μg/ml	Significantly improved sperm motility, membrane integrity, acrosome integrity, and mitochondrial function	(62)
		Carboxymethyl cellulose	0.25% (w/v)	Relatively higher sperm motility, acrosome integrity, and proportion of sperm with high mitochondrial membrane potential	(132)
		Selenium nanoparticles (Se-NPs)	1 μg/ml	Significantly improved sperm motility, total motility, progressive motility, and plasma membrane integrity	(133)
	Japanese white rabbit	AFP III	1 μg/ml	Significantly increased proportion of rapidly motile sperm	(63)
	Horse	Graphene oxide (GO)	0.01 wt%	Post-freeze-thaw sperm motility increased from 24.3% to 71.3%	(120)
	Pig	AFPs	1–40 mg/ml	Enhanced low-temperature tolerance	(115)
	Bull	Selenium nanoparticles (Se-NPs)	1.0 μg/ml	Improved post-thaw sperm quality, reduced apoptosis	(74)
Oocyte	Mouse	L-proline oligomer (L-Pro8)	40mg/ml	Cryosurvival rate 99.11%; CPA concentration reduced from 4.3 M to 2.5 M; improved mitochondrial function	(10)
		AFP III	500 ng/ml	Post-thaw survival rate 94.6% vs 84.5%; blastocyst rate 89.1% vs 68.9%	(65)
	Bovine	AFGP8	1 mM	Significantly improved post-freezing oocyte survival rate (77.1% vs 67.4%) and fertilization ability	(66)
		Arctic yeast-derived LeIBP	0.1 mg/ml	Reduced ROS levels; maintained normal mitochondrial distribution and function; inhibited apoptosis	(67)
	Pig	AFGPs	40 mg/ml	Post-thaw survival rate 25% (0% in control group)	(64)
Embryo	Mouse	Arctic yeast-derived LeIBP	10 mg/ml	Increased intact follicle ratio after ovarian cryopreservation	(118)
	Zebrafish	Gold nanorods (GNRs) + Laser rewarming	1.2 × 10^18^ particles/m^3^	Laser rewarming group survival rates: 31%, 17%, 10% (1, 3, 24 h); 0% in control group	(134)
	Japanese white rabbit	AFP III	500 ng/ml	Embryo survival rate 80.7%, significantly higher than control group 68.2%	(63)
	Bovine	Exopolysaccharide ID1	100 μg/ml	Significantly improved post-thaw blastocyst re-expansion and hatching rates; restored BAX gene expression	(98)
Kidney (Organ)	Rat	Silica-coated iron oxide nanoparticles (sIONP) + VS55	10 mg Fe/ml	Nanoparticles perfusable and washable; no obvious damage after rewarming	(123)
Heart (Organ)	Rat	SPIONs + Custodiol HTK	10 mg Fe/ml	Uniform perfusion and washout achievable; no visible damage after 1-week liquid nitrogen storage and nanowarming	(135)

Trehalose, a well-recognized non-toxic cryoprotectant, holds significant potential in the field of cryopreservation. Although studies have explored intracellular trehalose delivery via techniques such as microinjection (138) and nanomaterials (139) to achieve cryopreservation independent of permeable protectants (e.g., DMSO or EG), its application in the semen cryopreservation of small ruminants (such as sheep and goats) remains relatively limited. It is typically used only as a non-permeating protectant in combination with low concentrations of glycerol. Its protective efficacy varies across different species (e.g., sheep, pigs) and studies, leading to some controversy. Furthermore, related research has largely focused on sperm and certain somatic cells, with insufficient exploration of their standalone application (137). Additionally, when combined with external magnetic field heating technologies (e.g., those based on Fe3O4 or gold nanoparticles), nanomaterials can also improve thawing efficiency. However, current methods are mostly confined to low-throughput laboratory settings; the complex preparation and high cost of nanomaterials still present a gap for practical application in livestock production. Moreover, current research predominantly centers on single-cell systems, while the cryopreservation of multicellular systems, and even entire organs, remains a significant challenge. Future studies should explore cryopreservation techniques at the tissue, organ, and organism levels to accelerate advancements in gene therapy, cell therapy, regenerative medicine, and assisted reproduction. In summary, with the rapid development of chemistry, material synthesis, and engineering technologies, advanced and efficient cryopreservation techniques will continue to meet the demands of production and scientific research, offering broad prospects for the preservation of excellent livestock germplasm resources. By systematically reviewing relevant progress, this paper aims to provide a reference for developing safe, high-quality, and efficient cryopreservation strategies, promoting their translation and application in livestock production.
